# Anaplastic Spindle Cell Squamous Carcinoma Arising from Tall Cell Variant Papillary Carcinoma of the Thyroid Gland: A Case Report and Review of the Literature

**DOI:** 10.1155/2017/4581626

**Published:** 2017-04-06

**Authors:** Darren K. Patten, Alia Ahmed, Owain Greaves, Roberto Dina, Rashpal Flora, Neil Tolley

**Affiliations:** ^1^Department of Surgery, Hammersmith Hospital, Imperial College Healthcare NHS Trust, London, UK; ^2^Department of Surgery and Cancer, The Imperial Centre for Translational and Experimental Medicine, Imperial College London, Hammersmith Campus, London, UK; ^3^Department of General Medicine, Wexham Park Hospital, NHS Frimley Health Foundation Trust, London, UK; ^4^Department of Life Sciences, Imperial College London, London, UK; ^5^Department of Histopathology, Hammersmith Hospital, Imperial College Healthcare NHS Trust, London, UK

## Abstract

Tall cell variant (TCV) of papillary thyroid carcinoma (PTC), an aggressive form of thyroid cancer, is characterised by 50% of cells with height that is three times greater than the width. Very rarely, some of these cancers can progress to spindle cell squamous carcinoma (SCSC) resulting in cancers with elements of both SCSC and TCV PTC. Here we report a case of SCSC arising from TCV PTC. In addition to this case, we have performed a literature review and compiled all published reports of SCSC arising from TCV PTC, including the nature of treatment and the prognosis for each of the 20 patients recorded. This is intended for use as a guide for clinicians in what the most appropriate treatment options may be for a newly diagnosed patient. Due to the rarity coupled with diagnosis occurring at a very advanced stage of disease progression, performing clinical trials is difficult and therefore drawing conclusions on optimal treatment methods remains a challenge.

## 1. Introduction

First described by Hawk and Hazard in 1976, tall cell variant (TCV) of papillary thyroid carcinoma (PTC) is defined as an aggressive thyroid tumour, with 50 per cent of cells having height at least two or three times greater than width and bearing nuclear characteristics of PTC [1, 2]. Interestingly, some cases of TCV PTC progress to spindle cell squamous carcinoma (SCSC) which is a rare form of anaplastic carcinoma, consisting of both spindle cell elements and squamous islands with focal keratinization [3, 4]. TCV PTC associated with SCSC can be divided into three types as described by Gopal et al. [5]. We present a rare case of type 1 anaplastic SCSC arising from TCV PTC, highlighting the diagnostic challenges, as well as a review of the literature of all reported type 1 cases.

## 2. Case

**Figure 1:**
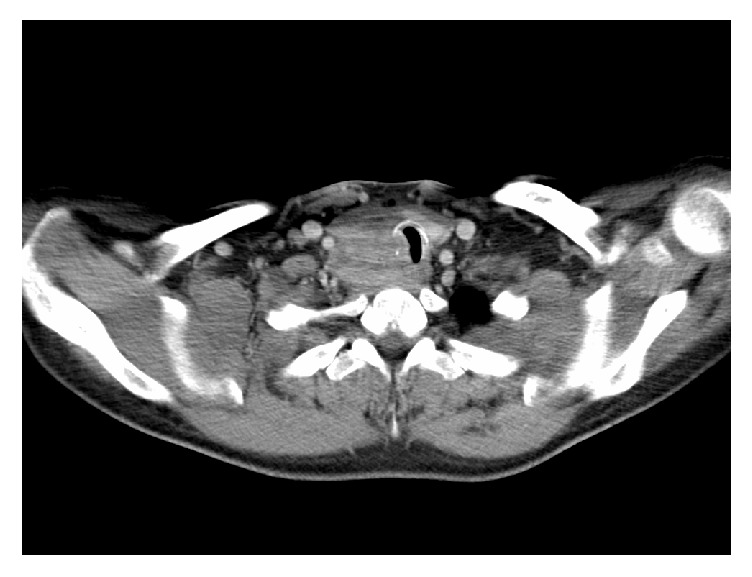
Neck CT scan showing the invasion of the trachea.

**Figure 2:**
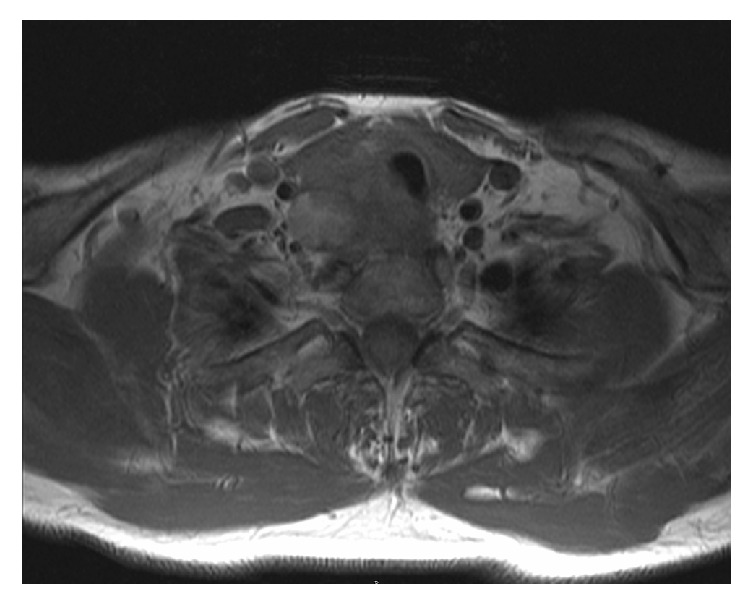
T1 MRI of neck showing invasion of trachea.

**Figure 3:**
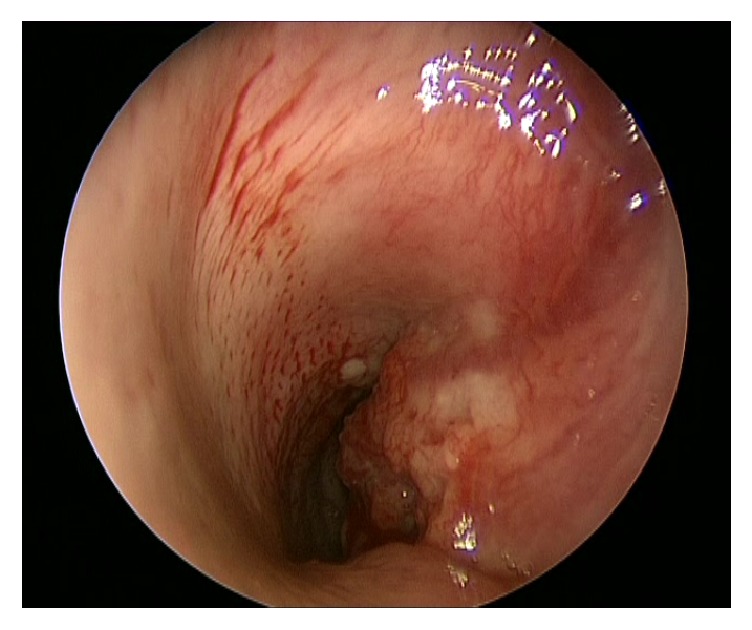
Tracheoscopy showing invasion of trachea from thyroid tumour.

In April 2011, a 51-year-old man presented to his primary care physician with symptoms of a mild sore throat and haemoptysis. Antibiotics were commenced for a presumed bacterial infection. He presented again two months later with shortness of breath and stridor and was referred to an Otolaryngologist for further care. Clinically, the patient was euthyroid and examination of the neck revealed a right-sided thyroid swelling with no lymphadenopathy. Blood tests were performed, including full blood count, thyroid function, calcium, phosphate, and vitamin D levels, and were all normal. The patient subsequently underwent an ultrasound scan (USS) and fine needle aspiration of the thyroid gland and was prescribed a short course of oral prednisolone. The results of the fine needle aspiration cytology (FNAC) suggested papillary carcinoma of the thyroid. Following steroid treatment, the patient was experiencing worsening haemoptysis, shortness of breath, and stridor associated with dizziness. The initial computer tomography (CT) scan of his neck revealed a mass extending from the posterior aspect of the right thyroid lobe, and further CT and magnetic resonance imaging (MRI) scan of the neck demonstrated that the thyroid mass had eroded into the trachea (Figures 1 and 2). Oral dexamethasone was commenced and the patient was referred to Hammersmith Hospital. A tracheoscopy was performed and vertical intraluminal tumour involvement was measured at 5 cm (Figure 3). The patient had no known drug allergies and his past medical history included hypercholesterolaemia which was controlled with medication.

The patient underwent a total thyroidectomy in September 2011, manubrial split (with levels 6 and 7 node dissection), and tracheal resection. End-to-end tracheal anastomosis was achieved with insertion of a tracheal stent and formation of a tracheostomy. The right recurrent laryngeal nerve was sacrificed owing to extensive tumour infiltration. Intraoperatively, the resected trachea was sent for frozen section histological analysis. The superior and inferior margins of the tracheal resection were clear of tumour and the specimen was reported as a moderately to poorly differentiated squamous cell carcinoma of the thyroid invading and ulcerating the tracheal mucosa.

Although the patient was found to be hypocalcaemic postoperatively, he made an uncomplicated recovery and the tracheostomy was removed 14 days following surgery. He was discharged with calcium supplementation. The case was discussed at the Thyroid Cancer Multidisciplinary Team Meeting where a decision was made to offer the patient a course of chemoradiotherapy.

Unfortunately, the patient died 4 weeks following hospital discharge.

## 3. Materials and Methods

The literature review was performed using the PubMed database from 1961 to 2012. The terms “thyroid” and “thyroid gland” were used in conjunction with “tall cell variant papillary carcinoma” and/or “squamous cell carcinoma” or “spindle cell squamous cell carcinoma.” A total of 162 articles were generated from the search of which only articles with cases documenting SCSC arising from TCV were included. Of the 162 articles, 3 were identified and a total number of 19 cases of documented SCSC arising from TCV PTC were included (Supplementary Table 1 in Supplementary Material available online at https://doi.org/10.1155/2017/4581626).

## 4. Histology 

**Figure 4:**
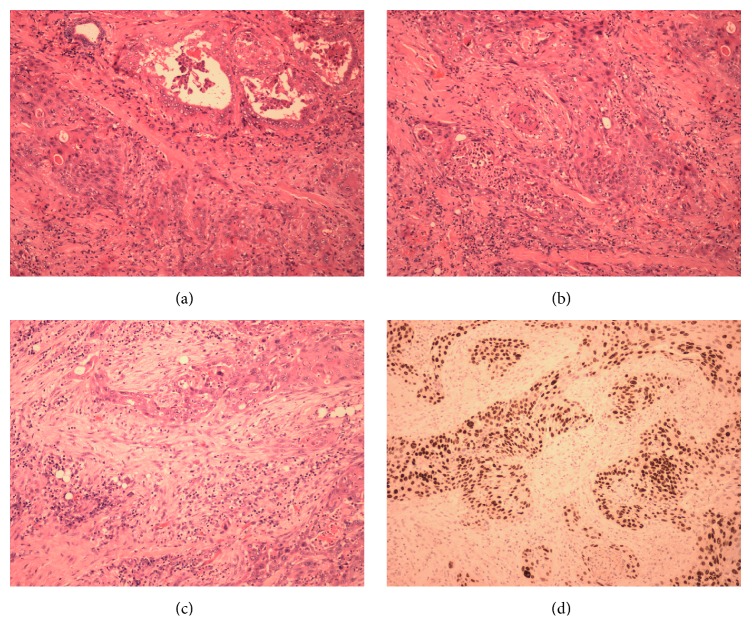
Histology of the tumour. (a) Features of papillary carcinoma including papillary architecture (with tall, well-formed papillae) and tumour cells that were columnar in shape, with nuclei showing overlapping, clearing, and pseudoinclusions. (b) Moderately to poorly differentiated squamous cell carcinoma. (c) Moderately to poorly differentiated squamous cell carcinoma with spindle cell elements. (d) Positive for (nuclear) thyroid transcription factor-1 (TTF-1) and Galectin-3 (cytoplasmic) in both components.

The thyroid contained a 5 cm tumour which showed two distinct morphologies. Part of the tumour (which was predominantly centred in the thyroid) showed features of papillary carcinoma including papillary architecture (with tall, well-formed papillae) and tumour cells that were columnar in shape, with nuclei showing overlapping, clearing, and pseudoinclusions (Figure 4(a)). As the length of the tumour cells was more than twice the width, the appearances were interpreted as those of the tall cell variant (of papillary carcinoma).

In addition, approximately 60–70% of the tumour showed features of moderately to poorly differentiated squamous cell carcinoma (Figure 4(b)) with spindle cell elements (Figure 4(c)).

The two components were intimately admixed and there were areas of transition from the papillary component to the squamous component.

Immunohistochemical analysis showed positivity for (nuclear) thyroid transcription factor-1 (TTF-1) and Galectin-3 (cytoplasmic) in both components (Figure 4(d)). Thyroglobulin was expressed in the papillary component, but not the squamous/spindle cell component. P63 was positive in the squamous component. The Ki67 proliferation index varied between 5% in the papillary component and 40% in the squamous component.

Extensive extrathyroidal extension was noted with invasion of the trachea and skeletal muscle. There was also widespread lymphovascular invasion and metastatic tumour was present in three lymph nodes.

## 5. Discussion

Thyroid carcinoma, being the most common endocrine malignancy, has an overall estimated incidence of 7.7 per 100,000.

TCV PTC is an aggressive tumour characterised by its tall columnar shape, with a height : width ratio of 2-3 : 1 and abundant eosinophilic or oxyphilic cytoplasm [1, 2, 6, 7]. Although a rare occurrence, TCV PTC may transform into anaplastic SCSC. TCV PTC is associated with adverse prognostic features including large tumour size, extrathyroidal extension, and vascular invasion, with a high incidence of locoregional recurrence, distant metastasis, and shorter disease-free survival [6, 8–14]. In addition, TCV possesses a more aggressive phenotype than conventional PTC, independent of age, gender, and tumour size [15]. On close examination of the cell cycle regulatory proteins such as p27, Ki67 cyclin D1, and P53 and eukaryotic translation initiation factors 4E and 2 alpha expression, TCV exhibits a molecular profile which is comparable to thyroid tumours with an unfavourable prognosis [4, 16–19].

Three main types of anaplastic SCSC arising from TCV PTC have been described by Gopal et al. based on histological examination: type 1 is defined by the presence of both TCV and SCSC within the initial resection; type 2 occurs when the SCSC component arises as a recurrence or metastasis in patients with a known history of TCV; type 3 is defined as SCSC presenting as a primary laryngeal squamous cell carcinoma in patients with or without a known history of TCV [5].

This case presented some diagnostic difficulties. Ultrasound guided FNAC is a useful tool in the investigation workup of thyromegaly but has proven to be misleading in many studies [20–22]. The initial FNAC result, in this case, suggested PTC, which was misleading. When assessing the time course and severity of the patient's progressive symptoms coupled with the results of the CT and MRI scans, a working diagnosis of PTC becomes less likely. Secondly, the tracheal resection sent frozen section histological analysis revealed a diagnosis of moderately to poorly differentiated squamous cell carcinoma (SCC). This scenario implied that there was a greater possibility that the patient possessed two primary tumours (PTC and laryngeal SCC) as opposed to both tumour components arising from the thyroid gland.

The patient presented in this case was eventually defined as having the type 1 variant of anaplastic SCSC arising from TCV PTC owing to the histological assessment of the resected specimen which revealed both SCSC and TCV. Although TTF-1 reactivity has also been shown to be present in primary squamous tumours of the lung [23, 24], its positivity along with Galectin-3 within the SCSC component implies that the latter originated from the thyroid gland.

On careful review on the current literature documenting anaplastic SCSC arising from TCV PTC, only the case series presented by Gopal et al. [5] (14 cases), Saunders and Nayar [25] (1 case), and Johnson et al. [6] (4 cases) have cases that demonstrate type 1 variant of this rare disease. Although Gopal et al. present 18 cases of type 1 anaplastic SCSC arising from TCV PTC only 14 of these are documented to have an SCSC component along with the TCV component of tumour [5]. Of the 5 cases presented by Bronner and LiVolsi, only 4 fall within the type 1 scenario [3] (Supplementary Table 1).

The median age at presentation is 71 years with females being more affected than males (1.5 : 1, resp.) (Table 1). Of the 20 cases reviewed, 16 (80%) presented with a rapidly enlarging thyroid mass whereas 4 (20%) presented with a thyroid nodule. Thyroid profile status was difficult to ascertain owing to a lack of reporting. With regard to treatment, the patient in this case underwent a total thyroidectomy and another patient underwent completion thyroidectomy and adjuvant chemoradiotherapy; 90% (18/20) of cases did not have documented treatment regimes. Regarding outcome and follow-up, 1 patient of 20 was reported to be alive after 6 months and the patient in this case deceased 4 weeks following surgery; 18 of 20 cases (90%) did not have documented prognosis and follow-up. The lack of outcome reporting is due to the rarity as well as aggressiveness (with survival rates of 20% at one year) of this unusual type of thyroid tumour and therefore makes development of an optimal treatment strategy a definite challenge [26].

The difficulties highlighted by this case are, firstly, the diagnosis of type 1 SCSC arising from TCV PTV. This is essentially a histological diagnosis, and as shown in this case, the FNAC results can be unreliable. Secondly, the rarity of this type of thyroid carcinoma coupled with late presentation has not allowed for a treatment regime to be established. When considering other types of aggressive thyroid tumours (e.g., anaplastic thyroid carcinoma) and their treatment, it is possible to suggest the use of chemoradiation coupled following surgical resection [27]. Another problem in the identification of new treatments for the type of thyroid carcinoma reported in this case is the rarity and aggressiveness of the tumour, rendering difficulty in recruiting patients who are clinically suited to participate in clinical trials.

## 6. Conclusion

We present the 20th documented case of type 1 anaplastic SCSC arising from TCV PTC highlighting the challenges in the diagnostic workup and a thorough review of all cases in the medical literature. This extremely rare neoplastic phenomenon forms a very small percentage of thyroid carcinomas and with its rarity and highly advanced stage of disease progression at presentation, recruiting patients to participate in clinical trials will inevitably lead to poor response rates from conventional and even newly emerging treatment regimens.

## Supplementary Material

Supplementary Table 1. Highlighting the documented cases, including clinical features, of spindle cell squamous carcinoma (SCSC) arising from tall cell variant (TCV) of papillary thyroid carcinoma (PTC) . The table also includes the case presented in this article.

## Figures and Tables

**Table 1:** The table shows demographics, clinicopathologic features, treatment, outcome, and follow-up of patients with anaplastic SCSC arising from TCV PTC. (Data based on the 20 reported cases including present case.)

*Patient demographics & clinicopathologic features*	
Number of patients	20
Median age (years)	71
Male : female ratio	1 : 1.5
Thyroid swelling	80% (16/20)
Thyroid nodule(s)	20% (4/20)
*Thyroid profile*	
Euthyroid	5% (1/20)
Not reported	95% (19/20)
*Treatment *	
Surgery	5% (1/20)
Surgery + chemotherapy/radiotherapy	5% (1/20)
Not reported	90% (18/20)
*Outcome & follow-up*	
Alive	5% (1/20)
Death from tumour	5% (1/20)
Disease-free follow-up	6 months
Follow-up not reported	90% (18/20)
