# Supplementary material for: Anaplastic Spindle Cell Squamous Carcinoma Arising from Tall Cell Variant Papillary Carcinoma of the Thyroid Gland: A Case Report and Review of the Literature
Source: Case Rep Endocrinol. 2017 Apr 6;2017:4581626. doi: 10.1155/2017/4581626 (PMC5397626; doi:10.1155/2017/4581626)
Supplement: Supplementary file 1 — Supplementary Table 1. Highlighting the documented cases, including clinical features, of spindle cell squamous carcinoma (SCSC) arising from tall cell variant (TCV) of papillary thyroid carcinoma (PTC) . The table also includes the case presented in this article. [file 4581626.f1.doc]

| 51/M | Presenting with symptoms of sore throat progressing to stridor and haemoptysis. Right sided thyroid swelling noted. Patient euthyroid. CT/MRI scans exhibited right sided thyroid mass with no lymphadenopathy. | Extrathyroidal extension to right side of trachea, LVI | PTC | 5.0 cm tumour showing anaplastic SCSC (60-70%) arising from TCV PTC. The SCSC component exhibited TTF-1 positivity | TT, 5.0 cm tracheal resection and level 6, 7 LN dissection | Deceased 4 weeks following surgery | - | Present Case |
| --- | --- | --- | --- | --- | --- | --- | --- | --- |
| 59/M | Stridor, dysphagia, hoarseness, large left thyroid mass with compression of the trachea. History of astrocytoma 10 years previously and hemi-thyroidectomy for benign lesion 5 years previously. | Invasion into perithyroidal soft tissue, parathyroid and skeletal muscle, LVI, PNI | NR | Anaplastic SCSC arising from TCV PTC | NR | NR | NR | Gopal et al. [5] |
| 51/F | Left thyroid mass (discovered during evaluation for hypercalcaemia, parathyroid adenoma, post thyroidectomy, radioactive iodine). | Extrathyroidal extension, PNI | NR | Anaplastic SCSC arising from TCV PTC | NR | NR | NR | Gopal et al. [5] |
| 78/M | Thyroid nodule noted during evaluation/treatment for parathyroid adenoma. | LVI | NR | Anaplastic SCSC (25% of tumour) arising from TCV PTC with extensive necrosis | NR | NR | NR | Gopal et al. [5] |
| 54/M | Right thyroid mass compressing trachea. | Extensive extrathyroid spread into trachea, lymphatics, skeletal muscle, PNI | NR | PTC TCV and follicular variant and anaplastic transformation with spindle cell and squamous features | NR | NR | NR | Gopal et al. [5] |
| 56/F | Thyroid mass and tracheal compression. | Extrathyroidal extension | NR | Anaplastic SCSC arising from TCV PTC | NR | NR | NR | Gopal et al. [5] |
| 54/M | Thyroid mass | LVI | NR | Anaplastic SCSC arising from TCV PTC | NR | NR | NR | Gopal et al. [5] |
| 68/F | Thyroid nodule | Extensive extrathyroidal extension into soft tissue and skeletal muscle, LVI | NR | TCV PTC with anaplastic transformation to SCSC (<5%) | NR | NR | NR | Gopal et al. [5] |
| 57/M | Thyroid nodule | NR | NR | Anaplastic SCSC arising from TCV PTC | NR | NR | NR | Gopal et al. [5] |
| 76/F | Thyroid mass | Extrathyroidal extension and invasion of skeletal muscle | NR | Anaplastic SCSC arising from TCV PTC | NR | NR | NR | Gopal et al. [5] |
| 82/M | Hoarseness, enlarging right neck mass over 2 months, history of Grave's disease (post radioactive iodine). | Extrathyroidal spread | NR | Anaplastic SCSC arising in association with TVC PTC | NR | NR | NR | Gopal et al. [5] |
| 92/F | Dyspnoea, hoarseness for 2 years. USS neck - 4cm x 3.1cm of the right thyroid lobe displacing the trachea; right vocal cord paralysis. | Extrathyroidal + soft tissue spread + LVI | NR | TCV PTC with transformation to anaplastic SCSC (<5%) | NR | NR | NR | Gopal et al. [5] |
| 59/F | Thyroid mass, history of left breast cancer 8 years previously (RT in 1999). | Extensive extrathyroidal extension and invasion of skeletal muscle | NR | SCSC arising in association with TCV PTC | NR | NR | NR | Gopal et al. [5] |
| 85/F | Rapidly growing thyroid mass. | Extensive extrathyroidal extension | NR | Anaplastic SCSC arising in association with TCV PTC and Warthin’s like variant | NR | NR | NR | Gopal et al. [5] |
| 76/F | Shortness of breath, imaging shows rapidly growing thyroid/laryngeal mass. | Extrathyroidal extension to laryngotracheal region and metastases to regional LNs | NR | Anaplastic SCSC arising in association with TCV PTC with extensive necrosis | NR | NR | NR | Gopal et al. [5] |
| 51/M | Right thyroid lobectomy in 1976 for papillary carcinoma. Presents in 1994 with 2cm mass in remaining thyroid, then again in 1997 with 3cm mobile left infraclavicular mass. | Metastatic tumour in infraclavicular LN - no histology | Atypical spindle cells arranged singly and in papillary clusters with foci of squamous cell differentiation (CT FNA 1997) | TCV PTC with 2 small foci of anaplastic SCSC (thyroid mass , 1994); anaplastic SCSC (infraclavicular mass, 1997) | Completion thyroidectomy and radical neck dissection followed by oral I-131 ablative therapy (1994); resection of infraclavicular mass and radical neck dissection, EBRT and CTx (1997) | Alive | 6 months | Saunders & Nayar. [6] |
| 77/F | Thyroid nodule revealed papillary CA. Patient declined further treatment until 21 months later when the nodule had enlarged resulting in dysphagia. | NR | PTC | 6.0cm tumour showing SCSC anaplastic transformation from TCV PTC | NR | NR | NR | Bronner & LiVolsi [3] |
| 82/F | Vocal cord paralysis and dysphagia lead to detection of a thyroid mass. | NR | NR | 6.4cm tumour showing TCV PTC with SCSC anaplastic transformation | NR | NR | NR | Bronner & LiVolsi [3] |
| 78/F | 2 year history of thyroid mass which underwent rapid enlargement with vocal cord paralysis and dysphagia. | NR | NR | 8.0cm tumour showing TCV PTC with SCSC anaplastic transformation | TT | NR | NR | Bronner & LiVolsi [3] |
| 74/F | Patient diagnosed with infiltrating duct CA of the breast 2 year before the development of a rapidly enlarging thyroid mass. Review of the breast CA showed comedo-type intraductal with an epithelial nonspindle cell infiltrating component. | NR | NR | Biopsy only: TCV PTC with SCSC anaplastic transformation | NR | NR | NR | Bronner & LiVolsi [3] |
